# The conifer biomarkers dehydroabietic and abietic acids are widespread in Cyanobacteria

**DOI:** 10.1038/srep23436

**Published:** 2016-03-21

**Authors:** Maria Sofia Costa, Adriana Rego, Vitor Ramos, Tiago B. Afonso, Sara Freitas, Marco Preto, Viviana Lopes, Vitor Vasconcelos, Catarina Magalhães, Pedro N. Leão

**Affiliations:** 1 Interdisciplinary Centre of Marine and Environmental Research (CIIMAR/CIMAR), University of Porto, Rua dos Bragas, 289, 4050-123 Porto, Portugal; 2 Department of Biology, Faculty of Sciences, University of Porto Rua do Campo Alegre, 4169-007 Porto (Portugal)

## Abstract

Terpenes, a large family of natural products with important applications, are commonly associated with plants and fungi. The diterpenoids dehydroabietic and abietic acids are defense metabolites abundant in resin, and are used as biomarkers for conifer plants. We report here for the first time that the two diterpenoid acids are produced by members of several genera of cyanobacteria. Dehydroabietic acid was isolated from two cyanobacterial strains and its identity was confirmed spectroscopically. One or both of the diterpenoids were detected in the cells of phylogenetically diverse cyanobacteria belonging to four cyanobacterial ‘botanical orders’, from marine, estuarine and inland environments. Dehydroabietic acid was additionally found in culture supernatants. We investigated the natural role of the two resin acids in cyanobacteria using ecologically-relevant bioassays and found that the compounds inhibited the growth of a small coccoid cyanobacterium. The unexpected discovery of dehydroabietic and abietic acids in a wide range of cyanobacteria has implications for their use as plant biomarkers.

The terpene family of natural products comprises tens of thousands of distinct molecules that have been isolated mainly from plants and fungi^1^. The anticancer drug taxol^2^ and the antimalarial artemisinin^3^ are notable examples from the plant world. Most terpenes are thought to have defensive or signaling roles^4^. Dehydroabietanes and abietanes, in particular dehydroabietic and abietic acids (**1** and **2**, respectively, Fig. 1), are well-studied diterpenes produced by conifers, together with a variety of mono- and sesquiterpenes, as major components of resin^5^. In the event of a wound, the lower molecular weight C_10_ and C_15_ terpenes volatilize once the resin is exposed to the atmosphere and are thought to act as defense toxins^5^,^6^. The heavier abietanes and dehydroabietanes do not volatilize and some are prone to oxidative polymerization; these metabolites are therefore thought to be involved in sealing the wound, engulfing insects and trapping potentially pathogenic microorganisms^5^,^6^. In conifers, **2** is biosynthesized via cytochrome P450-mediated oxidations of abietadiene, which in turn results from cyclization of copalyl diphosphate (derived from geranylgeranyl diphosphate), catalyzed by abietadiene synthase^7^. Abietadiene can presumably be oxidized to dehydroabietadiene; the conversion of the latter metabolite to **1** via P450-mediated oxidations has been demonstrated^7^. Due to their natural abundance and persistence, abietic and dehydroabietic acids (as well as their oxidation products and some other abietanes) have been used extensively in paleobotany, paleoethnobotany and organic geochemistry as markers for conifers^8^,^9^.

Despite that the overwhelming majority of described terpene molecules are found in plants and fungi, a small number of terpenoids have been known for decades to be produced by bacteria. These include geosmin and 2-methylisoborneol (produced by actinobacteria, cyanobacteria and myxobacteria)^10^, hopanoids (widely distributed among Bacteria)^11^ as well as beta-carotene and xantophylls and the phytoyl moiety of chlorophylls, which are produced by cyanobacteria^12^. A relatively small number of other terpenes are known from cyanobacteria. The sesquiterpenes β-cyclcitral, germacrene D, 6,11-epoxyisodaucane, isodihydroagarofuran, eremophilone and the nor-carotenoid β-ionone have been found in cultures of a single strain, *Calothrix* PCC 7507^13^. Tolypodiol^14^ and noscomin^15^ are related diterpenoids from the nostocalean *Tolypothrix nodosa* HT-58-2 and *Nostoc commune* EAWAG 122b, respectively. Two norabietane diterpenoids have been reported from *Microcoleus lacustris*^16^. In addition, some cyanobacterial secondary metabolites reflect the incorporation of isoprenoid units into scaffolds originating from other biosynthetic routes^17^,^18^,^19^. The recent recognition that bacterial genomes (including those from cyanobacteria) are rich in terpene synthases^20^,^21^ suggests that a large diversity of terpenes is yet to be discovered from the bacterial kingdom.

As part of our ongoing studies on cyanobacterial secondary metabolite diversity, we have been exploring diverse strains of cyanobacteria from our in-house collection (LEGE Culture Collection) for their small-molecule constituents. We report herein the NMR (Nuclear Magnetic Resonance)-guided isolation of **1** in two marine strains and the LC-HRESIMS (Liquid Chromatography coupled to High Resolution Electrospray Ionization Mass Spectrometry) detection of **1** and **2** in an extended group of cyanobacteria, indicating that the diterpenoids are widely distributed among this group of organisms.

## Results

### NMR-guided isolation and structural elucidation of dehydroabietic acid (1) from Synechococcales cyanobacterium strain LEGE 10388

A crude organic extract (1.18 g) was obtained from repeated extraction of biomass from the colonial cyanobacterium strain LEGE 10388 with CH_2_Cl_2_/MeOH (2:1), and fractionated using normal-phase vacuum liquid chromatography (VLC). Upon ^1^H NMR inspection of the resulting fractions, a set of aromatic signals (Supplementary Fig. S1) was detected in a fraction eluting with *n*-hexane/EtOAc (3:2). These resonances were followed by ^1^H NMR analyses of fractions resulting from successive rounds of normal-phase chromatography, until compound **1** was obtained with sufficient purity for detailed structural analysis.

Structural elucidation of **1** was carried out using a combination of HRESIMS as well as 1D and 2D NMR data (Fig. 2a, Supplementary Text S1 and Supplementary Figs S2–S8). The identity of **1** was confirmed as dehydroabietic acid following comparison of the NMR spectra and HRMS data of the cyanobacterial natural product with a commercial standard (Fig. 2b,c and Supplementary Figs S9 and S10).

### NMR-guided isolation of 1 from *Plectonema* cf. *radiosum* LEGE 06105

**Figure 2:**
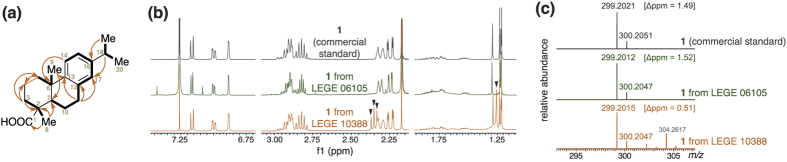
Isolation of dehydroabietic acid (**1**) from cyanobacteria. (**a**) Key HMBC (arrows) and COSY (thick bonds) correlations establishing that the planar structure of the metabolite isolated from the unidentified Synechococcales colonial cyanobacterium strain LEGE 10388 corresponds to that of **1**. (**b**) Comparison of the ^1^H NMR spectra in CDCl_3_ of a commercial standard of **1** (400 MHz) and of the metabolites purified from *Plectonema* cf. *radiosum* LEGE 06105 (400 MHz) and from Synechococcales cyanobacterium LEGE 10388 (400 MHz). Black triangles indicate peaks from a contaminant (see Methods section for details). (**c**) HRESIMS spectra (pseudomolecular ion clusters) of **1** (commercial standard) and of the metabolites purified from the two above-mentioned cyanobacteria; relative abundances are plotted for each spectrum.

In an independent effort, we were able to clearly identify the ^1^H NMR signature corresponding to the aromatic region of a dehydroabietane in a fraction obtained from a crude organic extract of the filamentous *Plectonema* cf. *radiosum* LEGE 06105. A combination of normal-phase column chromatographies and reversed-phase semipreparative and analytical HPLC allowed the purification of **1** from this strain (see Supplementary Fig. S11 for ^1^H NMR spectrum). The identity of the isolated natural product was confirmed by comparison of ^1^H NMR and HRMS data to a commercially available standard (Fig. 2b,c and Supplementary Fig. S9).

### Detection of dehydroabietic and abietic acid in other cyanobacterial strains

**Figure 3:**
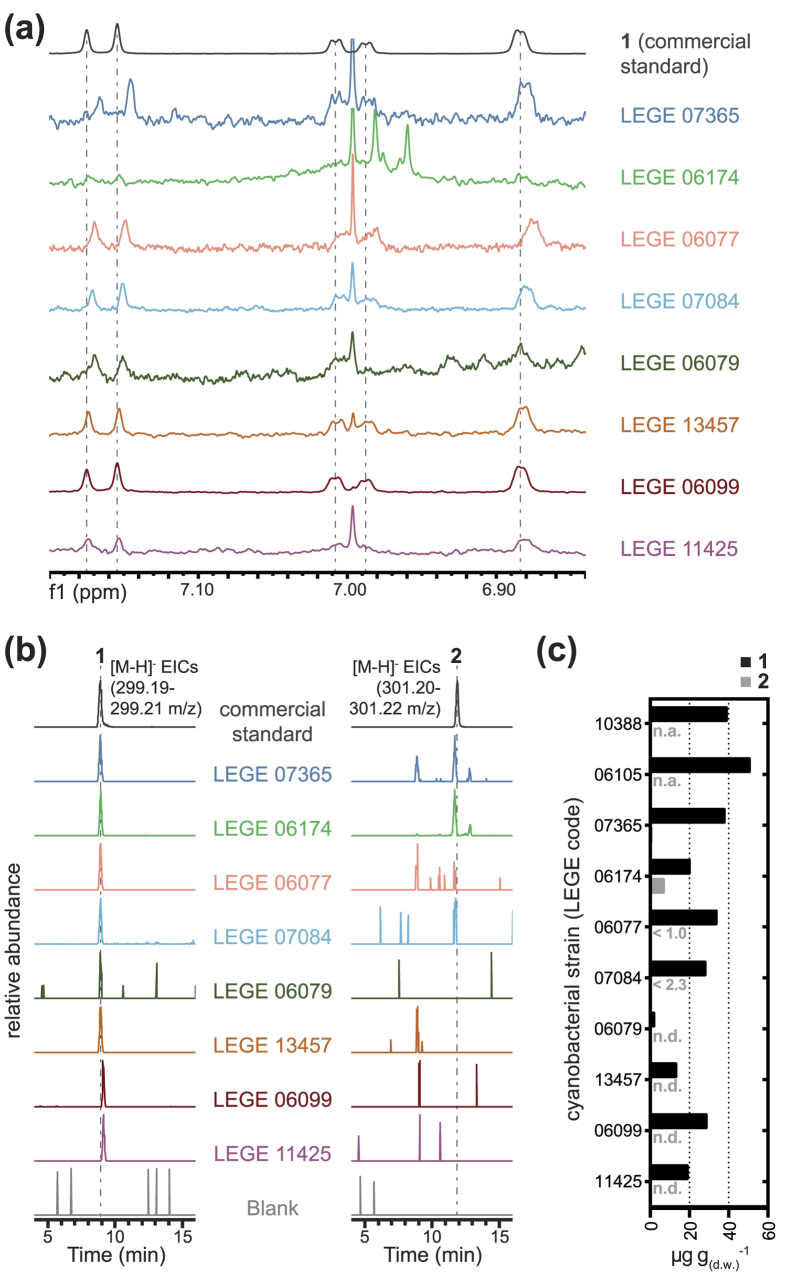
Detection of dehydroabietic (1) and abietic (2) acids in an extended group of cyanobacteria. Dehydroabietane-like aromatic proton signatures are observable (**a**) in chromatographic fractions originating from crude organic extracts of several cyanobacteria (strain codes indicated). LC-HRESIMS analysis of these fractions (**b**) confirms the presence of **1** and reveals that **2** is also found in some samples; relative abundances are plotted for each Extracted Ion Chromatogram (EIC). (**c**) LC-HRESIMS-based estimation (actual yields for strains LEGE 10388 and LEGE 06105) of the levels of **1** and **2** in the producing cyanobacteria (n.a., not analyzed; n.d., not detected; peak areas of **2** for strains LEGE 06077 and LEGE 07084 were below the lowest standard in the calibration curve and therefore the corresponding concentrations are reported as being lower than the value calculated from the standard with the lowest concentration).

Motivated by the discovery of **1** in two marine strains from our culture collection, we inspected chromatographic fractions from a set of 13 additional marine and freshwater cyanobacteria strains for ^1^H NMR aromatic signals consistent with the presence of **1**. Such fractions had been obtained from different cyanobacterial strains using the same methodology (organic extraction of the biomass followed by VLC) described above for the Synechococcales cyanobacterium unidentified strain LEGE 10388 and *Plectonema* cf. *radiosum* LEGE 06105. ^1^H NMR signals that could indicate the presence of **1** were found for fractions originating from eight other strains, of marine, estuarine and inland origins, namely *Nostoc* sp. LEGE 06077, *Nostoc* sp. LEGE 07365, *Chroococcidiopsis* sp. LEGE 06174, *Synechocystis* sp. LEGE 06079, *Synechocystis salina* LEGE 06099, *Leptolyngbya ectocarpi* LEGE 11425, *Leptolyngbya* sp. LEGE 07084 and *Nodosilinea* sp. LEGE 13457 (Fig. 3a). These fractions were submitted to LC-HRESIMS analysis which revealed that **1** was in fact present in all the samples (Fig. 3b). We could also detect, albeit with much lower intensity, a peak in some of the samples (*Nostoc* spp. strains LEGE 06077 and LEGE 07365, *Chroococcidiopsis* sp. LEGE 06174 and *Nodosilinea* sp. LEGE 07084) that, in all likelihood, corresponds to abietic acid (Fig. 3b). Estimates of the concentrations of **1** and **2** were calculated from LC-HRESIMS calibration curves or from actual purification yields and ranged from 1–50 μg/g (d.w.) for **1** and <1–7 μg/g (d.w.) for **2** (Fig. 3c). No ^1^H NMR aromatic signals indicative of the presence of **1** were found in VLC fractions and neither **1** or **2** were detected by LC-HRESIMS in crude organic extracts of the remaining five strains that were screened (Supplementary Fig. S12).

### Phylogenetic diversity of dehydroabietic acid producing cyanobacteria

**Figure 4:**
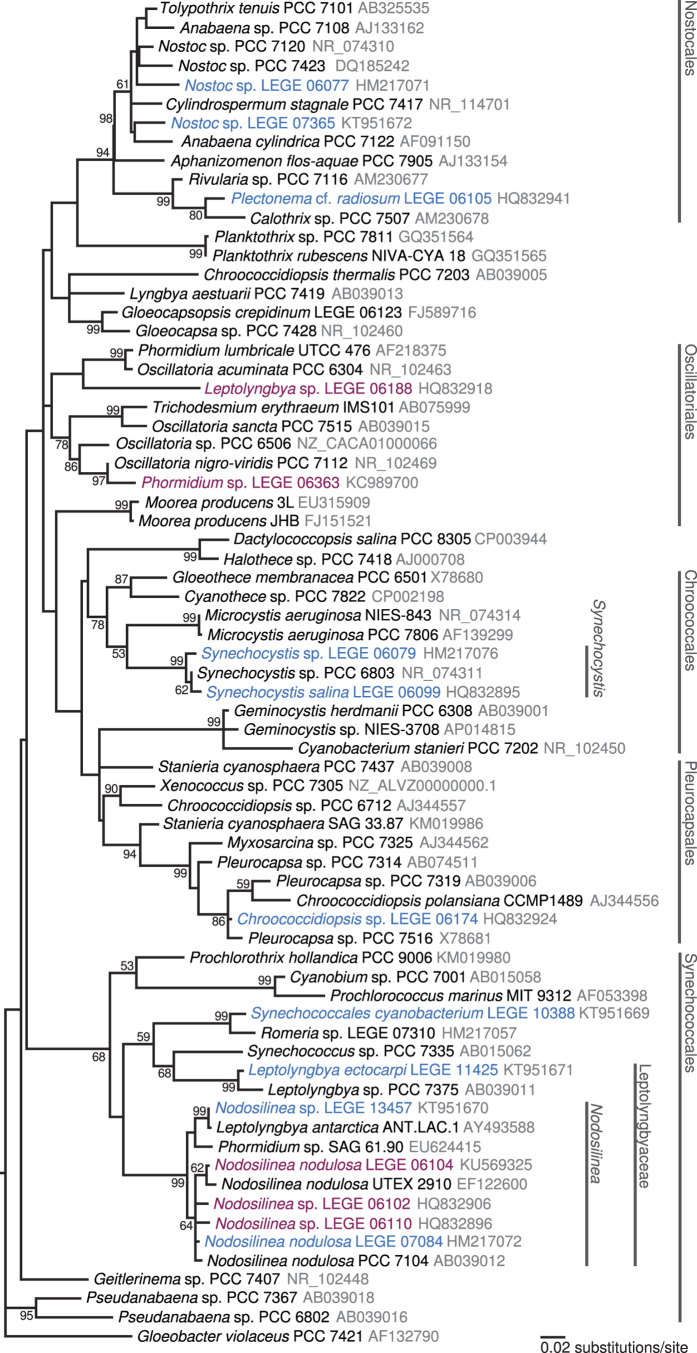
16S rRNA gene Maximum Likelihood (ML) phylogenetic tree of the phylum Cyanobacteria depicting the diversity of studied strains for which dehydroabietic acid (**1**) was (blue) or was not (purple) detected. *Chloroflexus aurantiacus* strain J-10-fl was used as an outrgroup (removed for clarity). Bootstrap values (1000 replicates) lower than 50 are omitted. Taxonomic levels higher than genus are according to Komárek *et al.*^64^ and Guiry & Guiry^65^.

To better understand the diversity of the strains in our culture collection for which we detected the production of **1**, a phylogenetic analysis was carried out with the objective of providing an overview of the phylum Cyanobacteria as well as the placement of the screened strains within it. Dehydroabietic acid-producers were found to be distributed throughout the cyanobacterial evolutionary tree, as expected from their morphological diversity (Fig. 4 and Supplementary Fig. S13). In particular, these are found in different branches within the Nostocales lineage (three strains), the “Leptolyngbyaceae family” clade (two different *Nodosilinea* strains and a red-pigmented *Leptolyngbya ectocarpi* LEGE 11425), and also within the *Synechocystis* lineage and the order Pleurocapsales clade (Fig. 4). Strains for which **1** was not detected were placed within the Synechococcales (all from the genus *Nodosilinea*) and Oscillatoriales lineages.

### Bioactivity of the diterpenoid acids towards aquatic organisms

**Figure 5:**
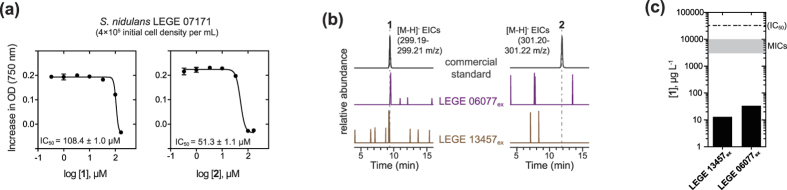
Dehydroabietic acid inhibits the growth of a marine coccoid cyanobacterium and is released into the medium by actively growing cyanobacteria. (**a**) Dose-response curves for exposure of the marine cyanobacterium *Synechococcus* cf. *nidulans* LEGE 07171 to **1** and **2**. (**b**) LC-HRESIMS analyses reveal the presence of **1** in culture supernatants of the cyanobacteria *Nostoc* sp. LEGE 06077 and *Nodosilinea* sp. LEGE 13457; relative abundances are plotted for each Extracted Ion Chromatogram (EIC). (**c**) LC-HRESIM-based estimation of the concentration of **1** in culture supernatants and comparison with concentrations for which inhibitory activity was observed towards *S. nidulans* LEGE 07171 (i.e., IC_50_ value and the shaded area corresponds to a range between the Minimum Inhibitory Concentrations with significant (P < 0.05, t-test) differences to the control treatment from two independent experiments −3 and 10 μg mL^−1^).

We tested the most abundant **1** for activity towards organisms that may interact with cyanobacteria in the natural setting. In particular, we evaluated the compound for antialgal and anticyanobacterial activity as well as for toxicity towards small crustaceans, which are known to feed on cyanobacteria^22^. When the dehydroabietic acid-producing *S. salina* LEGE 06099, the green microalga *Chlorella vulgaris* and the crustacean *Artemia salina* were exposed to concentrations of **1** up to 50 μg mL^−1^ (167 μM), no inhibitory or stimulatory effects on growth were observed (data not shown). However, the marine picocyanobacterium *Synechococcus* cf. *nidulans* LEGE 07171 was inhibited by **1** (Fig. 5a). Given this observation, we tested **2** for its anticyanobacterial or antialgal activity. Abietic acid was slightly more potent than **1** towards *S. nidulans* LEGE 07171 (51.3 *vs.* 108.4 μM IC_50_ values, respectively, Fig. 5a), but was also not active towards *S. salina* LEGE 06099 and *C. vulgaris* up to 50 μg mL^−1^ (data not shown). To clarify whether the anticyanobacterial interaction observed could have a relevant ecological meaning, we investigated whether the two terpenoids were released to the culture medium by actively growing cultures of cyanobacteria. We analyzed supernatants from two of the producing strains, *Nostoc* sp. LEGE 06077 and *Nodosilinea* sp. LEGE 13457, using LC-HRESIMS and detected only **1**, which was also the most abundant compound inside the cells (Fig. 5b). Estimation of the concentration of **1** in the supernatants indicates that it is two orders of magnitude lower than the minimum inhibitory concentration (MIC) observed in the *S. nidulans* LEGE 07171 assays (Fig. 5c and Supplementary Fig. S14).

## Discussion

Our findings indicate that **1**–and most likely **2** (which we detected in very low amounts in our samples by LC-HRESIMS)–are widespread in cyanobacteria. This is, to our knowledge, the first report of any of these compounds among bacteria. Apart from vascular plants, **1** has been reported from a epiphytic lichen and its mycobiont^23^, as well as from the fungus *Armillaria mellea*, which is parasitic on several conifers^24^. One immediate implication of the present report is that the suitability of **1** and **2** as biomarkers for plants needs to be reassessed. The two diterpenoid acids are used extensively as higher plant biomarkers in paleobotanical and geochemical studies^25^, and while being present in different plant or plant-derived materials^26^, they are most commonly associated with evergreen gymnosperms (in particular conifers) and their resin^25^,^27^. The validity of the large majority of studies that used **1** and **2** as markers for conifers is not called into question by the present discovery, mainly because other biomarkers originating from different biosynthetic routes are commonly used in conjunction with the resin acids^25^. Also, the amount of **1** and **2** of plant origin in studied materials is likely to be much higher than that of cyanobacterial origin, as cyanobacteria do not often reach high cell densities in the environment (blooms in aquatic systems being a notable exception e.g.^28^). Nevertheless, the cyanobacterial origin for the two diterpenoids needs to be taken into account especially when important extrapolation is based on the detection of these metabolites. Given the large number of terpene synthases found in cyanobacteria^20^,^21^, it is possible that other terpenoid plant biomarkers are produced by these prokaryotes. In fact, retene–presumably derived diagenetically from **2**–was detected in marine-derived sediments from the Lower Paleozoic, before coniferous plants had evolved^29^. A subsequent study reported the GC (Gas Chromatography)-MS-based detection of retene in pyrolysates (300 °C) of the eukaryotic microalga *Chlorella protothecoides* and of the model cyanobacterium *Synechocystis* sp. PCC 6803^30^. The authors suggest that retene could derive from the thermal degradation of **2**, since two degradation intermediates (dehydroabietin and 1,2,3,4-tetrahydroretene) were tentatively identified by GC-MS analysis^30^. Interestingly, the dehydroabietic acid producers *S. salina* LEGE 06099 and *Synechocystis* sp. LEGE 06079 are closely related to *Synechocystis* sp. PCC 6803 (Fig. 4), but we were unable to detect **2** in these cyanobacteria. The previously reported cyanobacterial diterpenoids noscomin^15^, tolypodiol^14^ and the two norabietane diterpenoids from *Microcoleus lacustris*^16^ further illustrate the potential for diterpene production by cyanobacteria.

From our phylogenetic analysis, it is clear that the distribution of resin acid-producing cyanobacteria is transversal in the cyanobacterial phylum. We were unable to detect any of the the two resin acids in some strains, but it is possible that these were present at levels below the limit of detection. The small number of strains studied here restricts our ability to infer whether the present distribution is associated with horizontal gene transfer (HGT) events or if the production of **1** and **2** is ancestral, prior to the evolutionary radiation of these microorganisms. Cyanobacterial secondary metabolites such as microcystins and saxitoxins, for which production and biosynthetic potential have been evaluated exhaustively in a large number of cyanobacteria, are distributed among different genera (albeit not as widely as we report here for **1** and **2**) and have been shown to be inherited vertically^31^,^32^. Given the wide distribution and small sample size in our study, it is plausible that this is also the case for resin acid production in cyanobacteria.

A bacterial evolutionary origin for higher plant diterpene synthases is likely, based on their sequence homology with bacterial terpene synthases^33^. Conifer resin acids originate in the methylerythritol (MEP) pathway^5^,^34^ which is also the only known terpene biosynthesis pathway in cyanobacteria and employs the photosynthesis products pyruvate and glyceraldehyde-3-phosphate as substrates^12^. In fact, MEP-pathway biosynthetic events, including abietadiene synthesis and the following transformations that lead to **1** and **2** take place in plastids^5^, which have a cyanobacterial evolutionary origin^35^. Overall, these observations could suggest that resin acid production has been transferred from cyanobacteria to plants. In an attempt to support this hypothesis, and since over a hundred cyanobacterial genomes (including that of the putative abietic acid producer *Synechocystis* sp. PCC 6803) are available in public databases, we searched these for homologs of a conifer abietadiene synthase, but could not find any strong or widespread homologies, although hypothetical proteins with weak homology were identified (see Supplementary Methods and Supplementary Tables S1, S2 for details). Such absence of evident homology rather supports an eventual convergent evolution of cyanobacteria and conifer abietane biosynthesis. Still, in the plant kingdom, abietadiene-derived diterpenes are not exclusively found in gymnosperms–abietanes (including dehydroabietanes) have been found in different families of angiosperms (e.g. Lamiaceae^36^, Apiaceae^37^ and Ericaceae^38^). Therefore, a better understanding of the distribution of this group of metabolites (or of the genetic potential for their production) in both the bacterial and plant kingdoms will be instrumental for clarifying how their biosynthesis evolved.

The defensive roles of **1** and **2** in higher plants–in particular engulfing or trapping pathogenic microorganisms are plausible for cyanobacteria. As readily available materials, the two resin acids have been extensively studied for their biological properties, and showed activity (at concentrations above 5 μg mL^−1^) in antibacterial assays e.g.^39^,^40^,^41^. Metabolite **1** was also found to inhibit methanogenesis in different bacteria^42^, to have anti-ageing activity in *C. elegans*^43^, mild cytotoxicity against cancer cell lines^44^ and antifungal activity^40^. Compound **2** has shown mild cytotoxicity against cancer cells^45^, mid-micromolar inhibition of lipoxygenase activity^46^, and anticonvulsant activity^47^. Both **1** and **2** were genotoxic to marine organisms^48^,^49^. We have tried to complement this range of bioactivities by testing the secondary metabolites in a series of ecologically-relevant assays, namely using phyto- and zooplankton organisms. The growth inhibiting activity towards *S. nidulans* LEGE 07171 and the fact that the compounds are released to the medium by actively growing cyanobacteria suggests that **1** and **2** may act as allelochemicals. Still, MICs observed in the *S. nidulans* assay were much higher than the estimated levels of **1** in the culture media (Fig. 5) and, while *de facto* concentrations of allelochemicals near the producing cells can be orders of magnitude higher^50^, it is not clear at this time whether the observed allelopathic effect is relevant in the natural setting. The low levels of **1** in cyanobacterial cells and spent medium could be more consistent with a signaling role such as the defense signaling carried out by nanomolar levels of the diterpene WAF-1 ((11*E*,13*E*)-labda-11,13-diene-8α,15-diol) as a response to wounding in tobacco plants^51^ or those of the ubiquitous phytohormone diterpenes gibberelins^52^.

To conclude, by isolating metabolites from two cyanobacteria and elucidating their structures to reveal their identity as **1**, we have established that this compound is produced by cyanobacteria. Our subsequent investigation on the production of **1** and **2** in cyanobacteria brings up important questions regarding diterpene biosynthesis evolution at the bacterial and plant level. Future research should clarify whether the two metabolites are also frequent in other plants and algae. Access to the genes involved in the biosynthesis of the two resin acids in cyanobacteria will facilitate the study of their actual distribution. Also relevant will be to determine whether other biosynthetically-related resin acids (such as levopimaric acid, neoabietic acid and palustric acid) are found in cyanobacteria. Therefore, studies employing **1** and **2** as biomarkers need to not only take into account that these are not exclusive from the plant world but also consider the possibility that other diterpenes that are commonly associated with plants may have a (cyano) bacterial origin.

## Methods

### General procedures and materials

1D and 2D NMR data were acquired in either a 400 MHz Bruker Avance III or a 600 MHz Bruker Avance II HD equipped with a 5 mm cryoprobe. The samples were dissolved in deuterated chloroform (99.8% deuterium for fractions and 100% deuterium for pure compounds, Alfa Aesar).

All reagents were ACS grade quality or higher, unless stated otherwise. All solvents were ACS grade quality except for those used for HPLC sample preparation and separations, which were HPLC gradient grade.

### Cyanobacterial and algal strains and cultures

A list of the cyanobacterial strains used in this study for the prospection of **1** and **2** is depicted in Table 1. All cyanobacteria were grown under a light (~30 μmol photons m^−2^ s^−1^):dark cycle (14:10h) at 25 °C. Freshwater and estuarine strains were cultured in Z8 medium^53^. Marine strains were cultured in either Z8 medium supplemented with 25 g L^−1^ NaCl (strains LEGE 06099, LEGE 06174, LEGE 06104, LEGE 06102, LEGE 06110 and LEGE 06188), MN medium^54^ (strains LEGE 10388 and LEGE 06105), or Z8 medium supplemented with 25 g L^−1^ of synthetic sea salts (Tropic Marin) (strain LEGE 11425). The culture media used for the growth of marine strains were additionally supplemented with 20 μg L^−1^ of vitamin B_12_.

Stock cultures of cyanobacterial strains used as target organisms in biological assays (*Synechocystis salina* LEGE 06099 and *Synechococcus* cf. *nidulans* LEGE 07171) were maintained in Z8 medium supplemented with 25 g L^−1^ NaCl. The microalga *Chlorella vulgaris* LEGE Z-001, also used as a target organism, was cultured in Z8 medium. Light and temperature conditions for the biological assay target strains were the same as those detailed above for the other cyanobacterial strains.

### Isolation of 1 from Synechococcales cyanobacterium strain LEGE 10388

Lyophilized biomass (13.9 g) from multiple cultures of the cyanobacterium was repeatedly extracted by percolation with a warm (<40 °C) mixture of CH_2_Cl_2_/MeOH (2:1). The resulting crude organic extract (1.18 g) was subjected to vacuum liquid chromatography (VLC) using silica gel 60 (0.015–0.040 mm, Merck KGaA) as stationary phase and a step-wise mobile phase gradient from 100% n-hexane to 100% EtOAc and then to 100% MeOH, yielding ten fractions. One of these (fraction E, 38.9 mg, eluting with 60% EtOAc in hexane) was found by ^1^H NMR to contain a set of downfield signals of interest and was further fractionated using a silica gel SPE cartridge (Strata SI-1, 5 g, Phenomenex), using a step-wise gradient from 10% EtOAc in hexane to 100% EtOAc. Subfractions eluting with 20% EtOAc in hexane (total 4.7 mg) contained the ^1^H NMR peaks of interest, together with those corresponding to an aliphatic contaminant; these subfractions were pooled and subjected to another round of normal phase chromatography using an SPE cartridge (Strata SI-1, 2 g, 1% EtOAc in hexane to 100% EtOAc stepwise gradient), to yield **1** (0.5 mg, eluting with 12% EtOAc in hexane) with enough purity for detailed 1D and 2D NMR analyses. The contaminant was still present in this sample, but to a much lesser extent than in the previous subfraction and did not interfere with the structure elucidation process using 1D and 2D NMR data, due to minimal superimposition with signals from **1**.

### Isolation of 1 from *Plectonema* cf. *radiosum* LEGE 06105

The lyophilized biomass (9.5 g) obtained from *Plectonema* cf. *radiosum* LEGE 06105 was extracted repeatedly with a 2:1 mixture of CH_2_Cl_2_/MeOH and the resulting crude extract (0.70 g) fractionated using VLC, as detailed for the colonial Synechococcales strain LEGE 10388. Upon inspection of the VLC fractions by ^1^H NMR, signals consistent with the aromatic moiety of a dehydroabietane were present in fraction E (21.7 mg, eluting with 60% EtOAc in hexane), which was further separated on a SPE cartridge (Strata SI-1, 5 g, Phenomenex) as detailed above. The fraction containing the ^1^H NMR signals (2.4 mg, eluting with 20–25% EtOAc in hexane) was subjected to two rounds of analytical-scale HPLC purification using a Synergi Fusion-RP column (250 × 4.6 mm, 10 μ, Phenomenex). The initial separation was carried out under isocratic conditions at 99% MeCN (aq) with a flow of 1.2 mL min^−1^ and the peaks eluting within *t*_R_ = 1–7 min were re-injected into the HPLC system and separated isocratically using 75% MeCN (aq), to yield pure **1** (0.5 mg, *t*_R_ = 9.6 min).

### Detection of 1 and 2 in cyanobacteria-derived chromatographic fractions

Cyanobacterial biomass extraction and VLC fractionation, performed as described above, is routinely carried out in our laboratory as part of bioactive secondary metabolite isolation efforts e.g.^55^. We therefore maintain a small library consisting of solvent-free VLC fractions, stored at −80 °C.The ^1^H NMR spectra of fractions from our library originating from the strains depicted in Table 1 (with the exception of the unidentified Synechococcales cyanobacterium strain LEGE 10388 and *Plectonema* cf. *radiosum* LEGE 06105 from which **1** had already been isolated) were carefully inspected for the presence of the characteristic set of signals that originate from **1**–a doublet (*J* ~ 8 Hz), a doublet of doublets (*J* ~ 2, 8 Hz) and another doublet (*J* ~ 2 Hz) around δ7.16, δ7.00, and δ6.88 ppm, respectively. The fractions that contained such signals, as well as fractions of similar polarity (fractions D, E and F, eluting with 50%, 60% and 80% EtOAc in hexane, respectively) or crude extracts from the strains whose fractions did not contain the afore-mentioned aromatic signals were then submitted to LC-HRESIMS analysis. A portion of the material was dissolved in MeOH to a final concentration of 0.5 mg mL^−1^ (fractions) and 1 mg mL^−1^ (crude extracts) and injected (20 μL) into an HPLC system composed of an Accela PDA detector, Acella Autosampler and Acella 600 Pump (Thermo Fischer Scientific) to perform the chromatographic separation. For HRESIMS, the system was hyphenated with a LTQ Orbitrap XL hybrid mass spectrometer (Thermo Fischer Scientific) controlled by LTQ Tune Plus 2.5.5 and Xcalibur 2.1.0. A linear gradient from 40 to 100% MeCN (aq) over 30 minutes with a flow rate of 0.8 mL min^−1^ was used in conjunction with a Gemini C18 column (5 μm, 110 A, 4.6 mm ID × 150 mm, Phenomenex). The capillary voltage of the electrospray ionization source (ESI) was set to 3.0 kV. The capillary temperature was 300 °C. The sheath gas and auxiliary gas flow rate were at 40 and 10 (arbitrary unit as provided by the software settings). The capillary voltage was −48 V and the tube lens voltage −247.79 V. To estimate the concentrations of **1** and **2** in the samples, calibration curves were carried out in the above system and conditions, using serial dilutions of commercial standards of **1** (≥95%, Sigma Aldrich) and **2** (technical grade, ~75%, Sigma) in MeOH. Extracted Ion Chromatograms (299.20 ± 0.01 m/z for **1** and 301.21 ± 0.01 m/z for **2**) were used for the integration of the standard chromatographic peaks. The purity of the standard material was not taken into account for the estimations which, in the particular case of **2**, may have led to an overestimation of up to 33% of the levels of the metabolite in the samples. An unknown difference in LC-HRESIMS conditions between: i) injections of commercial standard **1** and of the samples featured in Figs 3b and 5b, and ii) all subsequent injections (including commercial standard **2**), led to a delay of ~0.8 min in retention times. For clear interpretation of Figs 3b and 5b,a subtraction of 0.8 min to the EIC of standard **2** was applied.

### Genomic DNA extraction, amplification and sequencing of the 16S rRNA gene

Total genomic DNA (gDNA) was isolated from fresh biomass of *Nodosilinea* sp. LEGE 06104, Synechococcales cyanobacterium LEGE 10388, *Nostoc* sp. LEGE 07365, *Leptolyngbya ectocarpi* LEGE 11425 and *Nodosilinea* sp. LEGE 13457, using a commercial kit (PureLink Genomic DNA Mini Kit, Thermo Fisher Scientific), according to the instructions provided for Gram-negative bacteria. The purified gDNA was inspected for integrity in an agarose gel stained with GelRed (Biotium) and was then used as a template for the amplification of a major portion of the 16S rRNA gene. PCR amplification was carried out as previously detailed^56^. Briefly, a Taq polymerase (GoTaq, Promega) was employed and amplicons were obtained by using the primer pairs 27F-781R and/or 359F-1494R^57^,^58^. The PCR products were purified (Cut & Spin columns, GRiSP), ligated to a pGEM-T Easy vector (Promega) and cloned into TOP10 cells (Thermo Fisher Scientific). Purified plasmids (GenElute Plasmid Miniprep Kit) were submitted for Sanger sequencing (GATC Biotech) using M13 primers. The resulting sequences were assembled and manually inspected for quality using the Geneious v8.0 software package (Biomatters Limited) to produce a consensus sequence of the 16S rRNA gene amplified from each cyanobacterium (GenBank accession numbers KT951669-KT951672, KU569325). The 16S rRNA gene sequences for the other cyanobacteria strains used in this study (Table 1) had been reported previously^59^,^60^,^61^.

### Phylogenetic analysis

The 16S rRNA gene sequences from all the cyanobacteria strains screened for the presence of **1** and **2** in this study, as well as publicly available sequences from selected (including several reference strains) unicellular, colonial, filamentous non-heterocystous and heterocystous cyanobacteria were used to construct a phylogenetic tree. An alignment of the 16S rRNA gene sequences from the 10 strains producing **1**, the 5 strains that were tested but for which **1** was not detected, the 56 selected cyanobacteria and from *Chloroflexus aurantiacus* strain J-10-fl (outgroup) was carried out using MAFFT through the Geneious v8.0 software package. The alignment was truncated to a core region (754bp). jModelTest2^62^ was used to select the best nucleotide substitution model, which was found to be TPM3uf + I + G from the corrected Akaike Information Criterion (AICc). Using this model and the MEGA6 package^63^, a maximum likelihood (ML) phylogenetic tree was constructed for the dataset (partial deletion, 95% threshold), with bootstrap analysis (1000 replicates) and topology optimization (NNI).

### Biological activity assays

Stock solutions of **1** and **2** up to 5 mg mL^−1^ were prepared in DMSO (cell culture grade, Sigma Aldrich) and tested at 1% (v/v) in anticyanobacterial, antialgal and also in toxicity assays with *Artemia salina* (only for compound **1**), which were carried out as detailed previously^56^. Briefly, fresh culture stocks of the cyanobacteria *Synechocystis salina* LEGE 06155, *Synechococcus* cf. *nidulans* LEGE 07171 and the green microalga *Chlorella vulgaris* LEGE Z-001 were diluted in fresh culture medium to an OD 750 nm (200 μL) of ~0.1 (measurement with the microplate lid on) and added (198 μL) to microplate wells containing two microliters (2 μL) of the stock solution of **1** or **2**. Optical densities (750 nm) were measured in a microplate reader (Synergi HT, BioTek) right after inoculation and following a 7-day exposure period. For the *A. salina* assay, aliquots (198 μL) of 15–20 freshly hatched nauplii (from dried cysts) in artificial seawater were added to the microplate wells that contained two microliters (2 μL) of the stock solution of **1**. Mortality rates were determined after 48h. An antibiotic cocktail (penicillin 5000 units mL^−1^, streptomycin 5 mg mL^−1^ and neomycin 10 mg mL^−1^, Sigma Aldrich), was used as positive control for cyanobacteria, and potassium dichromate (4 μg mL^−1^) was used as a positive control for the green microalga and *A. salina*, while DMSO (1%, v/v) was used as a negative control for all target organisms. When growth inhibition caused by **1** or **2** was observed, dose-response curves were produced and IC_50_ values calculated (bottom fit values corresponding to the positive control optical densities) using Prism 6 (GraphPad Software).

### Detection and estimation of 1 and 2 in culture supernatants

A *Nodosilinea* sp. LEGE 13457 culture growing at 19 °C in Z8 medium for 120 days and a *Nostoc* sp. LEGE 06077 growing at 25 °C in Z8 medium for 21 days were filtered through 0.22 μm polyethersulfone syringe filters. The spent media were colorless and transparent, and there was no macroscopic evidence of cell senescence or lysis in any of the cultures. One-hundred milliliters (100 mL) of each of the supernatants were then liquid-liquid extracted with 200 mL of EtOAc. For each sample, the solvent in the combined organic layers was removed under reduced pressure and the residue was weighed and dissolved in a known volume of MeOH (<2 mL). Each solution was filtered through a 0.22 μm syringe filter (GHP, Pall Corporation) to remove insoluble material, injected (20 μL) and analyzed in the LC-HRESIMS system described above under the same chromatographic and detection conditions described for the intracellular fractions. Estimation of the levels of **1** was also carried out using a calibration curve as detailed for VLC fractions.

## Additional Information

**How to cite this article**: Costa, M. S. *et al.* The conifer biomarkers dehydroabietic and abietic acids are widespread in Cyanobacteria. *Sci. Rep.* **6**, 23436; doi: 10.1038/srep23436 (2016).

## Supplementary Material

Supplementary Information

## Figures and Tables

**Figure 1:**
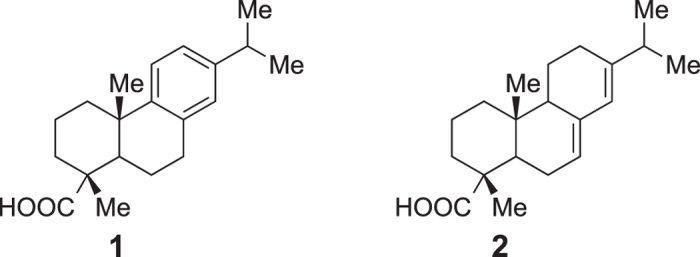
Structures of dehydroabietic acid (1) and abietic acid (2).

**Table 1:** Cyanobacterial strains used in this study for the detection of 1 and 2.

Taxon^a^	Strain code	Sampling location^b^	Habitat	Reference
unidentified colonial Synechococcales	LEGE 10388	Vila Nova de Mil Fontes	marine, intertidal	this study
*Plectonema* cf. *radiosum*	LEGE 06105	Praia da Luz	marine, intertidal, epiphytic on green macroalga	Brito *et al.*^59^
*Nostoc* sp.	LEGE 06077	Caminha (Minho river estuary)	estuarine, mesotidal, planktonic	Lopes *et al.*^60^
*Synechocystis* sp.	LEGE 06079	Vila Nova de Gaia (Douro river estuary)	estuarine, mesotidal, benthic	Lopes *et al.*^60^
*Nodosilinea nodulosa* (*Leptolyngbya* sp.)	LEGE 07084	Caminha (Minho river estuary)	estuarine, mesotidal, benthic	Lopes *et al.*^60^
*Synechocystis salina*	LEGE 06099	Moledo do Minho	marine, intertidal, tide pool, epilithic	Brito *et al.*^59^
*Nodosilinea* sp.	LEGE 06102	S. Bartolomeu do Mar	marine, tidepool, epilithic	Brito *et al.*^59^
*Nodosilinea nodulosa*	LEGE 06104	Praia da Luz	marine, tide pool, epilithic	this study
*Nodosilinea* sp.	LEGE 06110	Moledo	marine, tide pool, epilithic	Brito *et al.*^59^
*Chroococcidiopsis* sp.	LEGE 06174	Aguda	marine, coastal, plankton	Brito *et al.*^59^
*Leptolyngbya* sp.	LEGE 06188	Lavadores	marine, coastal, plankton	Brito *et al.*^59^
*Phormidium* sp.	LEGE 06363	wastewater treatment plant	biofilm on biological treatment tank outlet	Martins *et al.*^61^
*Nostoc* sp.	LEGE 07365	Caminha (Minho river estuary)	estuarine, mesotidal, epilithic	this study
*Leptolyngbya ectocarpi*	LEGE 11425	“Pêlo Negro”–subtidal rock formation outside Leixões Harbor, Matosinhos	marine, subtidal, benthic	this study
*Nodosilinea* sp. (=*Leptolyngbya antarctica*)	LEGE 13457	McMurdo Dry Valleys (Antarctica)	terrestrial, endolithic, from a sandstone	this study

^a^previously published taxa names (e.g. before a taxonomic revision) are indicated in brackets

^b^all locations in Portugal, unless otherwise indicated.
