# Supplementary material for: The conifer biomarkers dehydroabietic and abietic acids are widespread in Cyanobacteria
Source: Sci Rep. 2016 Mar 21;6:23436. doi: 10.1038/srep23436 (PMC4800451; doi:10.1038/srep23436)
Supplement: Supplementary Information [file srep23436-s1.pdf]

Supplementary Information

The conifer biomarkers dehydroabietic and abietic acids are widespread in Cyanobacteria

Maria Sofia Costa, Adriana Rego, Vitor Ramos, Tiago Afonso, Sara Freitas, Marco Preto, Viviana Lopes, Vitor Vasconcelos, Catarina Magalhães, Pedro N. Leão*

*corresponding author: pleao@ciimar.up.pt

List of contents: page

Isolation and structure elucidation of 1 from cyanobacterial strains

Fig. S1 – ^{1}H NMR-guided isolation of **1** from strains LEGE 10388 and LEGE 06105 S2

Text S1 – Structural elucidation of **1** from strain LEGE 10388. S3

Fig. S2 – Structural elucidation of **1** from strain LEGE 10388 S4

Fig. S3 – ^{1}H NMR spectrum (400 MHz, $CDCl_3$) of compound **1** isolated from strain LEGE 10388 S5

Fig. S4 – APT spectrum (100 MHz, $CDCl_3$) of compound **1** isolated from strain LEGE 10388 S6

Fig. S5 – Multiplicity-edited HSQC spectrum (400 MHz, $CDCl_3$) of compound **1** isolated from strain LEGE 10388 S7

Fig. S6 – HMBC (400 MHz, $CDCl_3$) spectrum of compound **1** isolated from strain LEGE 10388 S8

Fig. S7 – COSY (400 MHz, $CDCl_3$) spectrum of compound **1** isolated from strain LEGE 10388 S9

Fig. S8 – NOESY (400 MHz, $CDCl_3$) spectrum of compound **1** isolated from strain LEGE 10388 S10

Fig. S9 – Comparison of the ^{1}H NMR spectra in $CDCl_3$ of a commercial standard of **1** (400 MHz) with those of purified **1** isolated from strain LEGE 06105 (600 MHz) and from strain LEGE 10388 (400 MHz) S11

Fig. S10 – Comparison of the ^{13}C NMR spectra (100 MHz, $CDCl_3$) of a commercial standard of **1** with the APT spectrum (100 MHz, $CDCl_3$) of purified **1** obtained from strain LEGE 10388 S12

Fig. S11 – ^{1}H NMR (600 MHz, $CDCl_3$) spectrum of compound **1** isolated from strain LEGE 06105 S13

Detection of 1 and 2 in cyanobacterial extracts or fractions

Fig. S12 – Compounds **1** or **2** were not detected by NMR and LC-HRESIMS in some cyanobacterial strains. S14

Optical microphotographs of cyanobacterial strains

Fig. S13 – Optical microphotographs of the cyanobacteria used in this study that were found to produce metabolite **1** S15

Estimation of the concentration of 1 and 2 in cells and supernatants

Fig. S14 – LC-HRESIMS calibration curves used to estimate the concentrations of the resin acids **1** and **2** in cyanobacterial cells and cyanobacterial culture supernatants S16

Database searches for abietadiene synthase homologs in cyanobacterial genomes

Table S1 – Cyanobacterial homologs of an abietadiene synthase (AAK83563.1) from *Abies grandis* S17

Table S2 – Genomic context of the abietadiene synthase homolog (WP_041033013) from *Tolypothrix camylonemoides* S17

Supplementary Methods – Database searches for abietadiene synthase homologs in cyanobacterial genomes S18

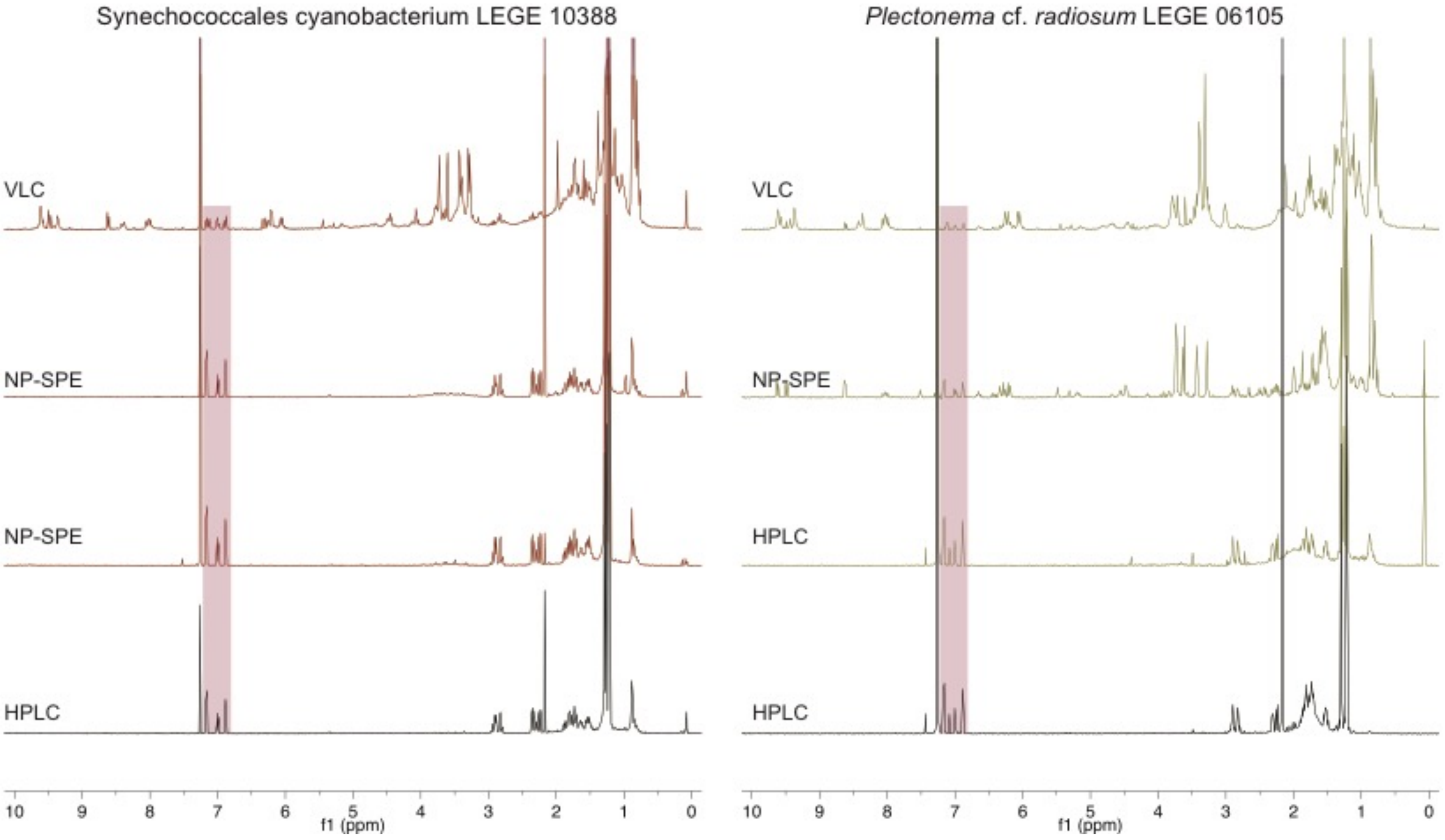

Fig. S1 – ^{1}H NMR-guided isolation of **1** from strains LEGE 10388 and LEGE 06105. ^{1}H NMR spectra ($CDCl_3$, 400 MHz) of fractions from sequential chromatographies (as annotated) leading to the isolation of **1** from both cyanobacteria. A set of downfield peaks (δ7.20-6.85) that guided the isolation is highlighted.

Supplementary Text 1 – Structural elucidation of **1** from strain LEGE 10388.

HRESIMS of **1** was consistent with a molecular formula of $C_{20}H_{28}O_2$ and seven degrees of unsaturation ($[M-H]^-$ 299.2015, Δppm = 0.5). ^{1}H NMR analysis (see Supplementary Fig. S3 for spectrum) of the aromatic region of the purified natural product revealed a 1,2,4-trisubstitued six-membered aromatic ring, as well as an upfield doublet (δ1.22, J = 6.9 Hz) for six protons, characteristic of an isopropyl moiety. Analysis of data from a ^{13}C APT (Supplementary Fig. S4) experiment promptly revealed the presence of a carbonyl at δ183.5, three aromatic fully substituted carbons at δ146.9, δ145.9 and δ134.8, three aromatic protonated carbons at δ127.1, δ124.3 and δ124.1, a quaternary carbon at δ47.5, an sp^3 methine at δ44.8 and three distinct resonances for methyl groups at δ25.3, δ24.1 and δ16.4. Other resonances corresponded to methylene groups or other eventual quaternary carbon(s). To further elucidate the structure of **1**, 2D NMR (HSQC, HMBC, COSY and NOESY) data were acquired (Supplementary Figs. S5-S8). HSQC data clarified the presence of another quaternary carbon at δ37.0, and the remaining carbon resonances were assigned to methylene groups. Extensive HMBC and COSY correlations quickly established a diterpene scaffold of the dehydroabietane type (Fig. 2a, Supplementary Fig. S2). One of the protons could not be accounted for in the NMR spectra, and was assigned to a carboxylic acid moiety, consistent with the C1 resonance of δ183.5. NOE correlations clarified relative stereochemistry (Supplementary Fig. S2).

a)

Carbon	δ_C[a]	δ_H, multiplicity (Hz)[b]	HMBC (^{1}H to indicated ^{13}C)	COSY	NOESY
1	183.5	-	-	-	
2	47.5	-	-	-	-
3	36.9	a:1.80 (m)	2, 4, 6	3b, 8	7
		b:1.71 (m)	4, 7	3a	8
4	18.7	1.76 (m)	6	-	5a
5	38.1	a:2.31 (m)	4, 6, 7	5b, 9	4, 5b, 9, 14, 15
		b:1.50 (m)	2, 3, 4	5a, 9	5a, 14
6	37.0	-	-	-	-
7	44.8	2.24 (dd, 12.5, 2.2)	1, 2, 3, 8, 9, 10, 11, 13	10a, 10b	3a, 5b, 11
8	16.4	1.29 (s)	1, 2, 3, 7, 9	3a	3b
9	25.3	1.22 (s)	6, 7, 8, 13	5a, 5b	5a, 14
10	21.9	a:1.86 (m)	6, 7, 11	7, 10b, 11	10b, 11
		b:1.54 (m)	6, 12	7, 10a, 11	10a, 11
11	30.1	2.89 (m)	7, 10, 12, 13, 17	10a, 10b, 14, 15, 17	7, 10a, 10b, 17
12	134.8	-	-	-	
13	146.9	-	-	-	
14	124.3	7.16 (d, 8.2)	6, 11, 12, 15, 16, 17	11, 15	5a, 5b, 9
15	124.1	7.00 (dd, 8.2, 2.0)	12, 13, 14, 17, 18	11, 14, 17	5a, 18, 19, 20
16	145.9	-	-	-	
17	127.1	6.88 (d, 2.0)	11, 13, 15, 18	11, 15	11, 19, 20
18	33.6	2.82 (sep, 6.9)	14, 16, 17, 19, 20	19, 20	15, 19, 20
19	24.1	1.22 (d, 6.9)	16, 18, 20	18	15, 17, 18
20	24.1	1.22 (d, 6.9)	16, 18, 19	18	15, 17, 18

[a]recorded at 100 MHz; [b]recorded at 400 MHz

b)

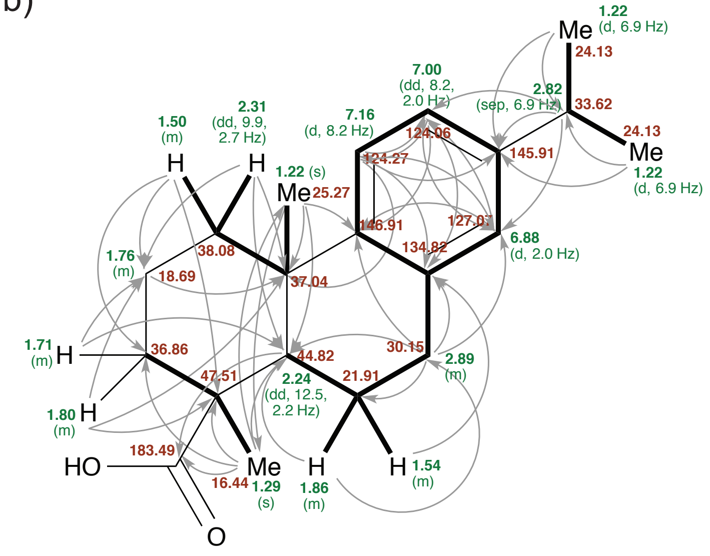

c)

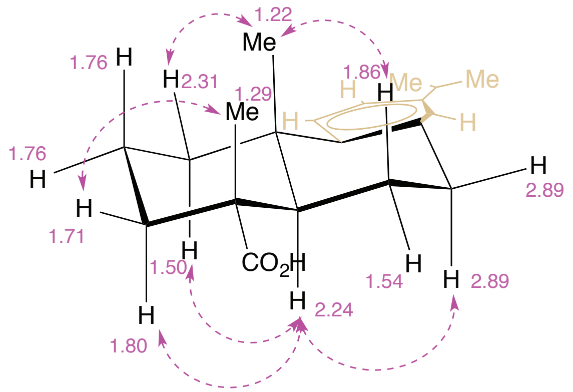

Fig. S2 – Structural elucidation of **1** from strain LEGE 10388. a) NMR data for **1** in $CDCl_3$; b) HMBC (arrows) and COSY (thick bonds) correlations extracted from the NMR data (^{13}C values are shown with two decimal places – as extracted from the spectra – but are only reproducible to one decimal place); c) Selected NOESY correlations confirming relative stereochemistry (assuming near-chair conformations).

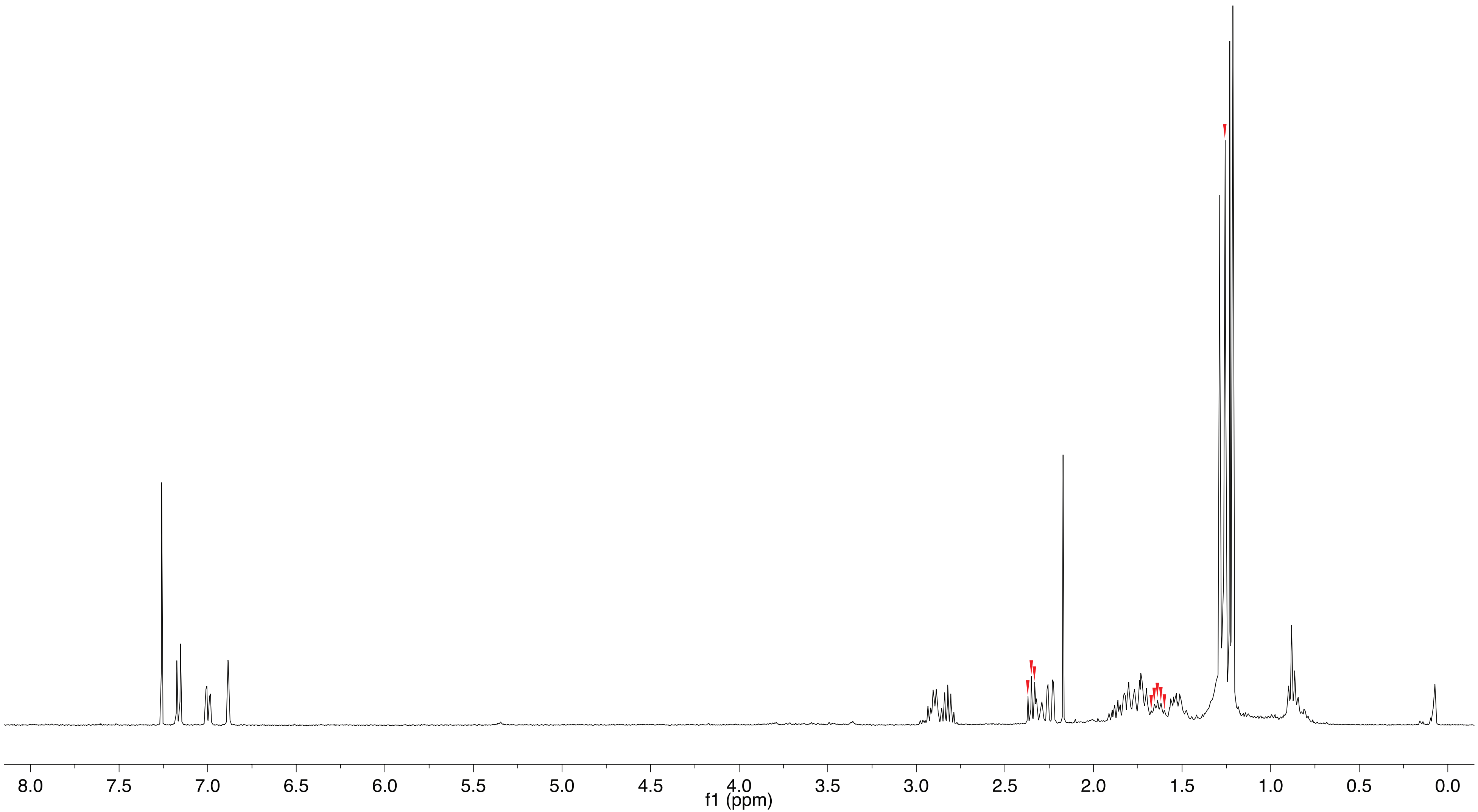

Fig. S3 – ^{1}H NMR spectrum (400 MHz, $CDCl_3$) of compound **1** isolated from strain LEGE 10388. Peaks corresponding to a fatty acid or fatty acylated impurity are annotated with red triangles.

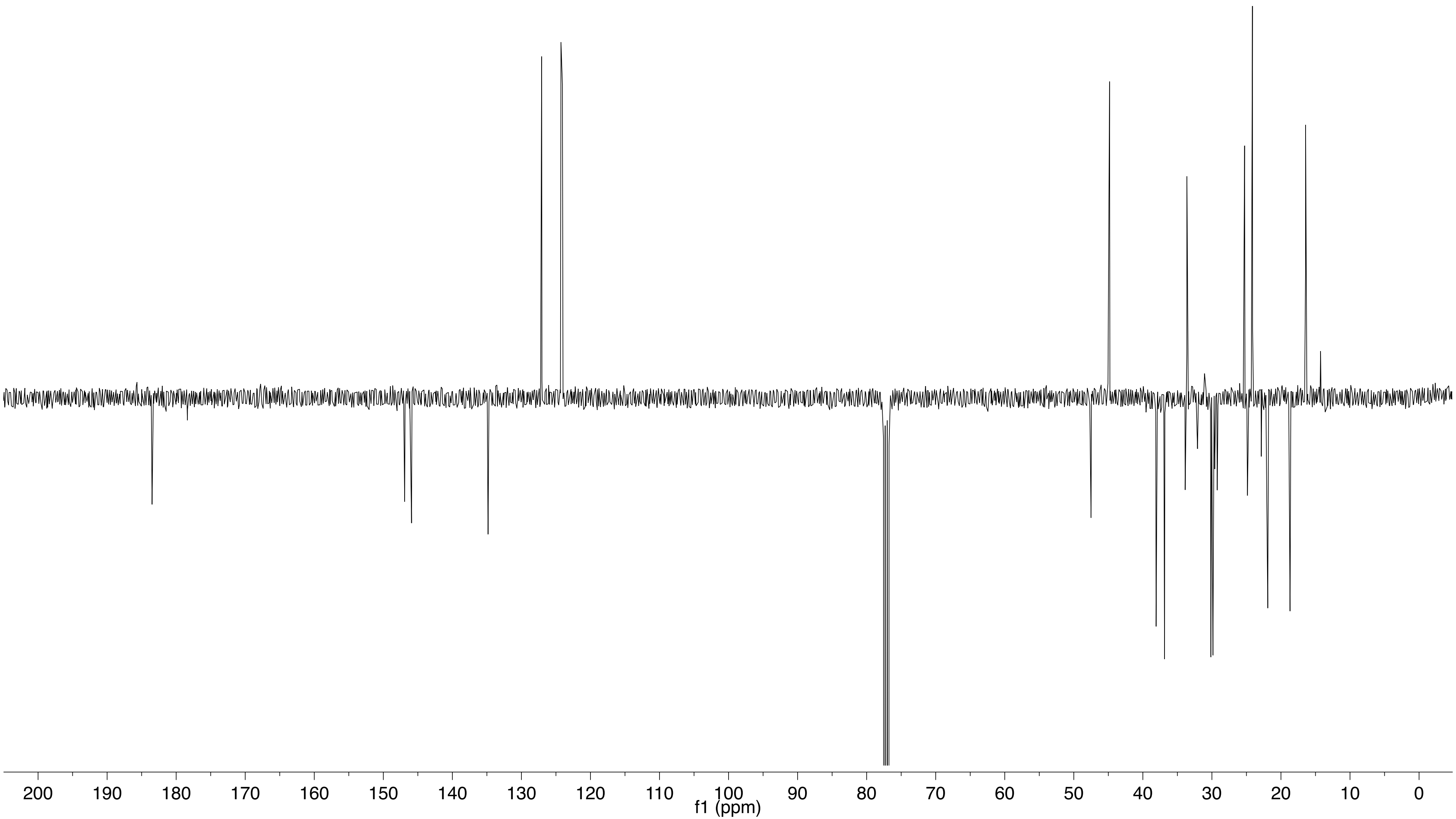

Fig. S4 – APT spectrum (100 MHz, $CDCl_3$) of compound **1** isolated from strain LEGE 10388.

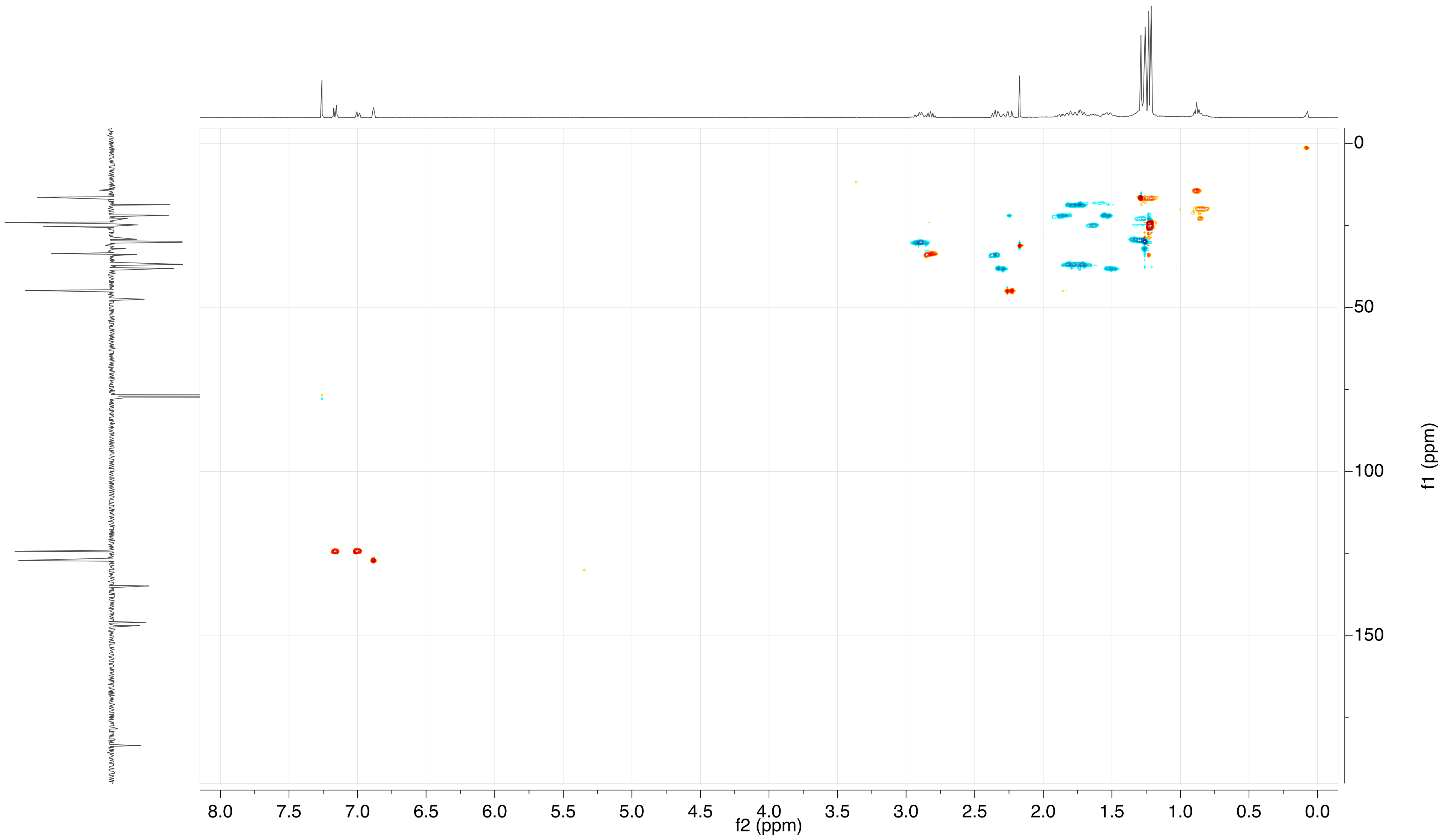

Fig. S5 – Multiplicity-edited HSQC spectrum (400 MHz, $CDCl_3$) of compound **1** isolated from strain LEGE 10388.

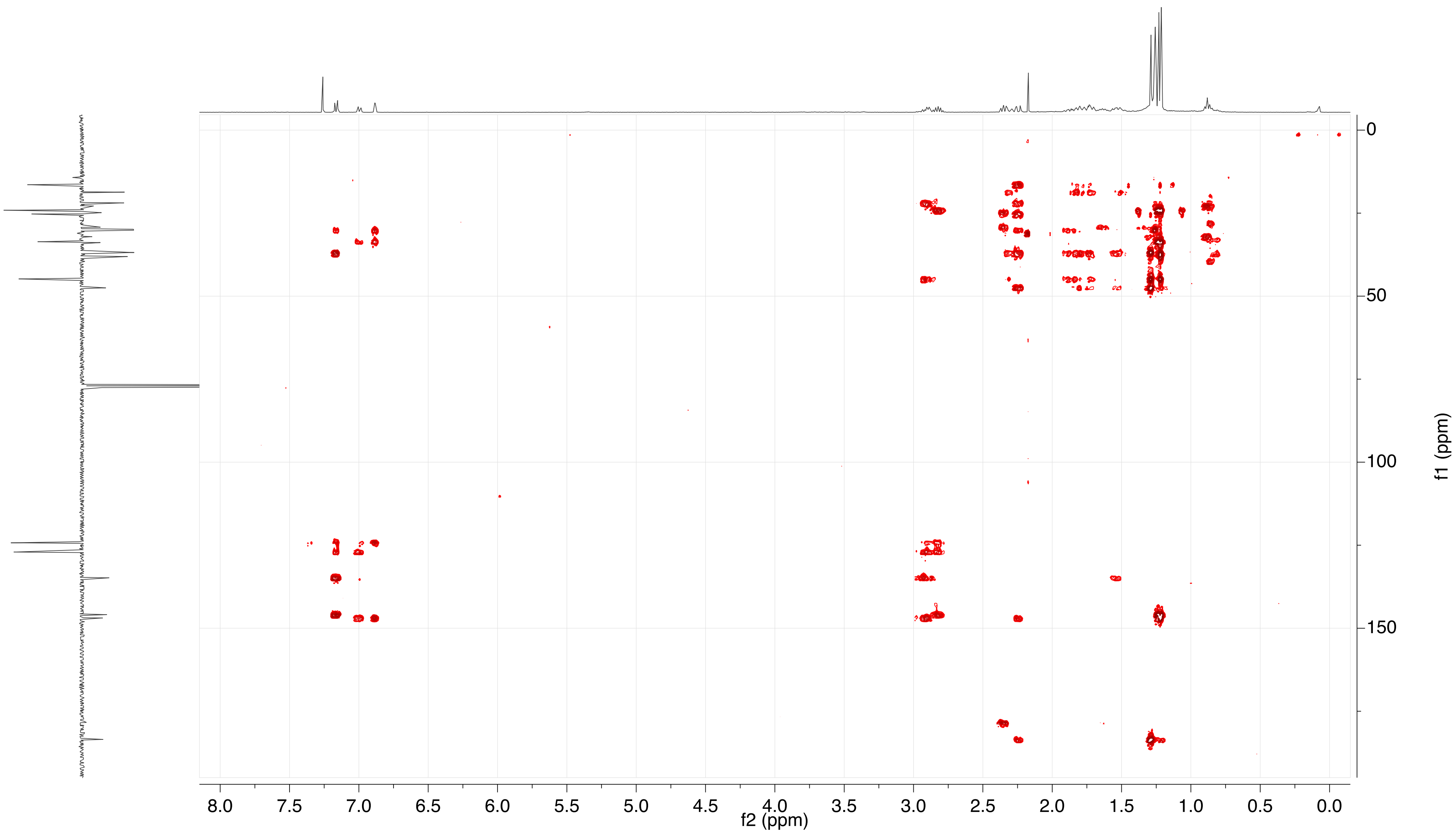

Fig. S6 – HMBC (400 MHz, $CDCl_3$) spectrum of compound **1** isolated from strain LEGE 10388.

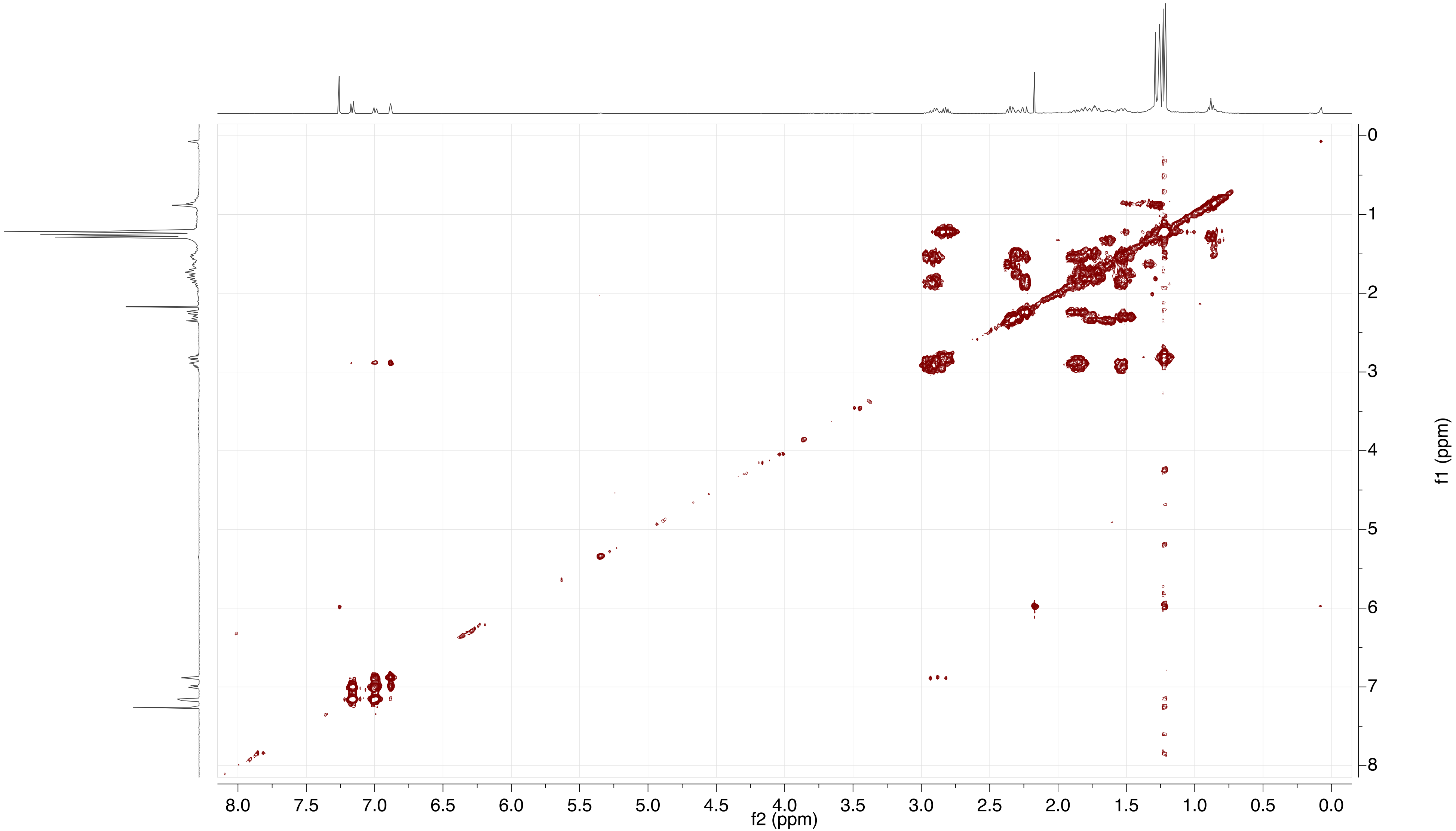

Fig. S7 – COSY (400 MHz, $CDCl_3$) spectrum of compound **1** isolated from strain LEGE 10388.

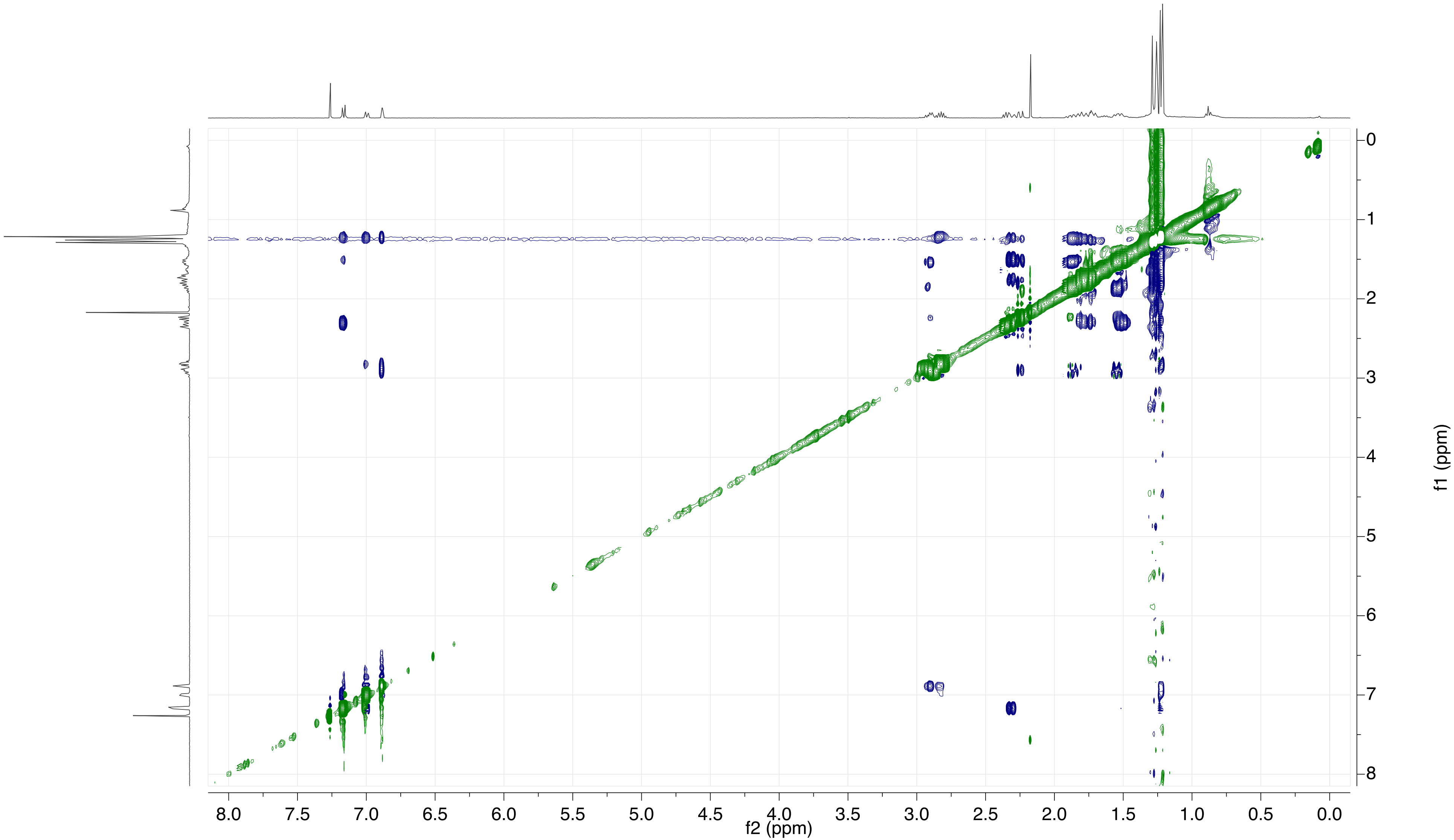

Fig. S8 – NOESY (400 MHz, $CDCl_3$) spectrum of compound **1** isolated from strain LEGE 10388.

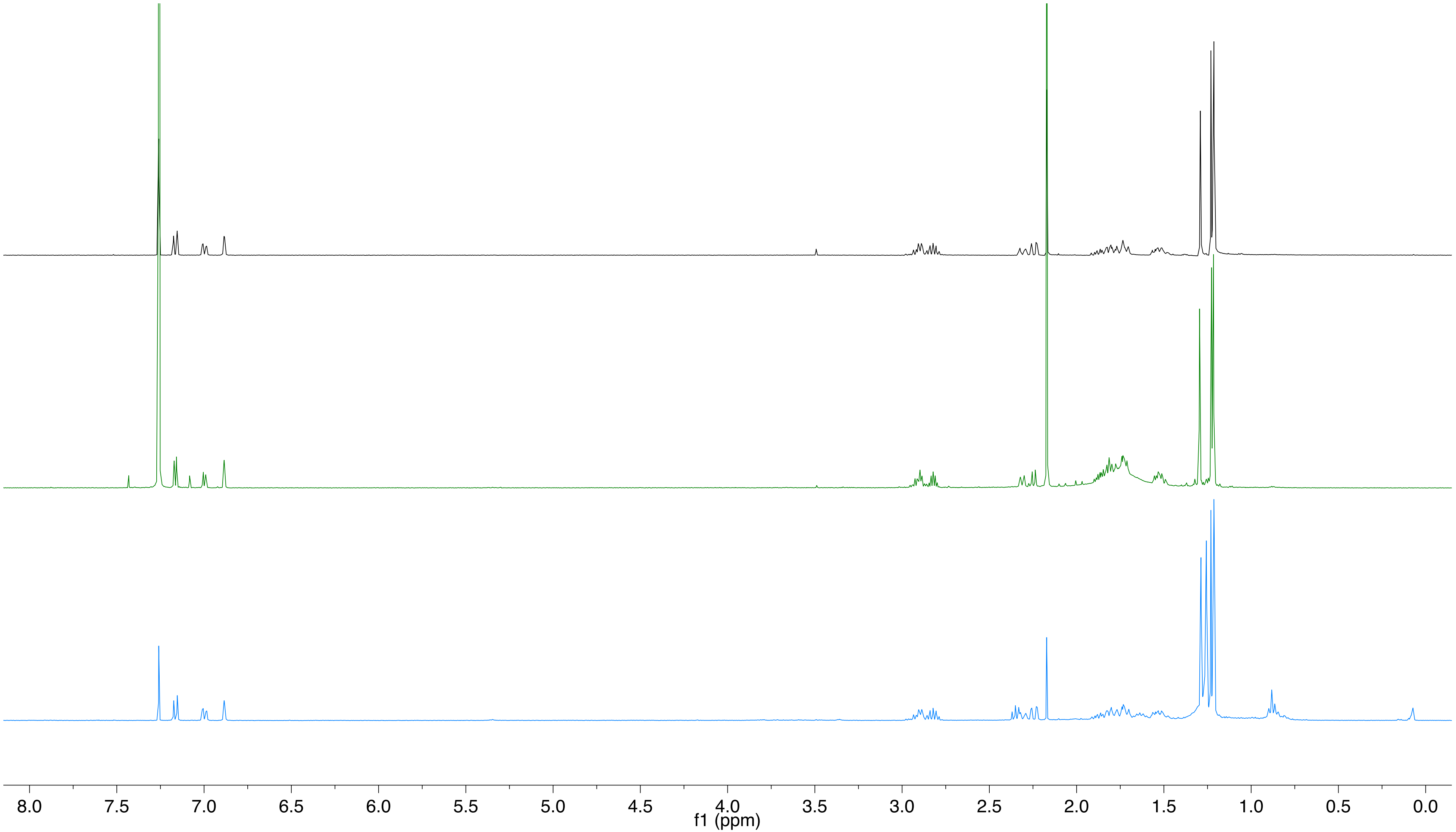

Fig. S9 – Comparison of the ^{1}H NMR spectra in $CDCl_3$ of a commercial standard of **1** (top, 400 MHz) with those of purified **1** isolated from strain LEGE 06105 (middle, 600 MHz) and from strain LEGE 10388 (bottom, 400 MHz).

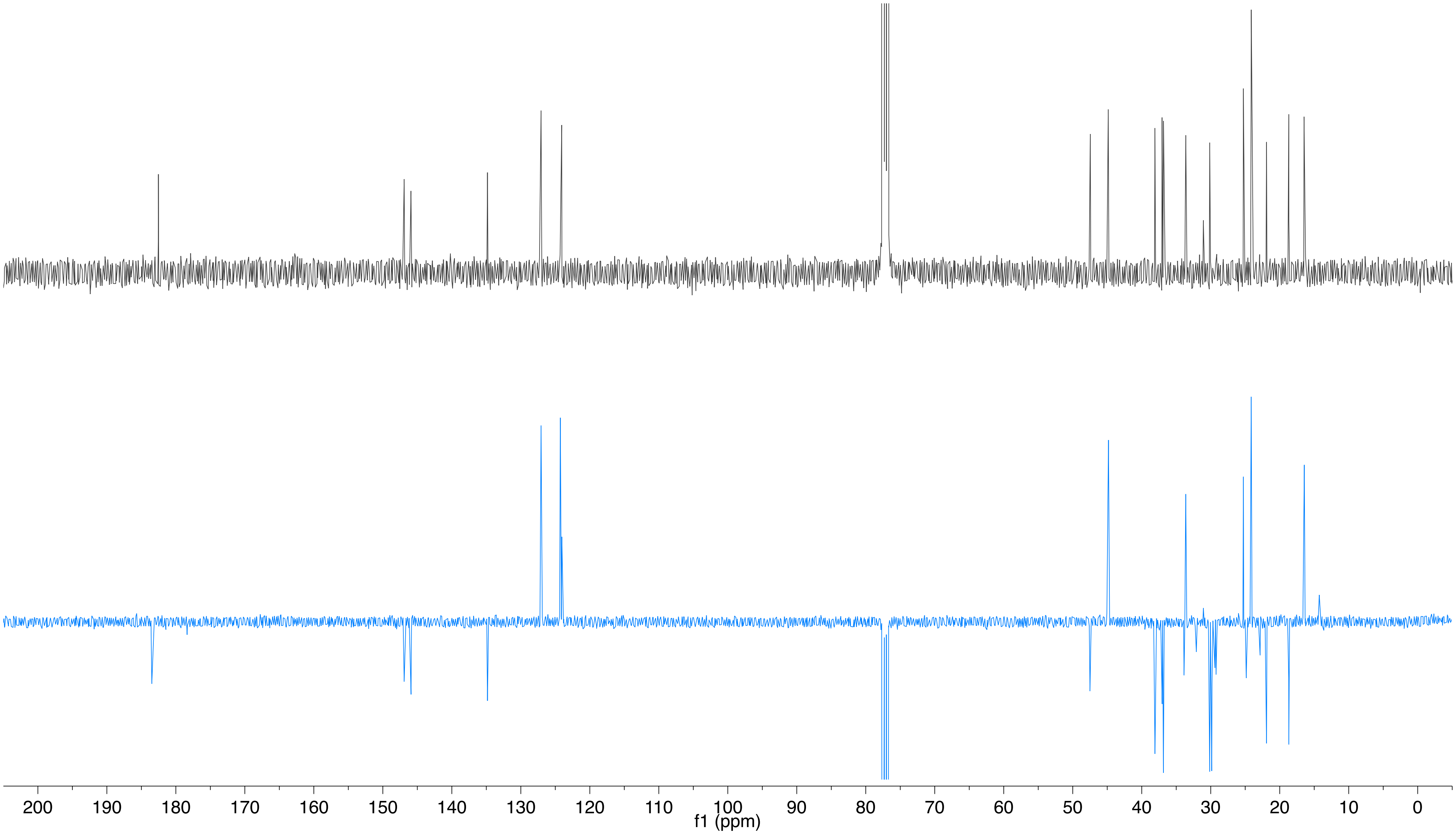

Fig. S10 – Comparison of the ^{13}C NMR spectra (100 MHz, $CDCl_3$) of a commercial standard of **1** (top) with the APT spectrum (100 MHz, $CDCl_3$) of purified **1** obtained from strain LEGE 10388 (bottom).

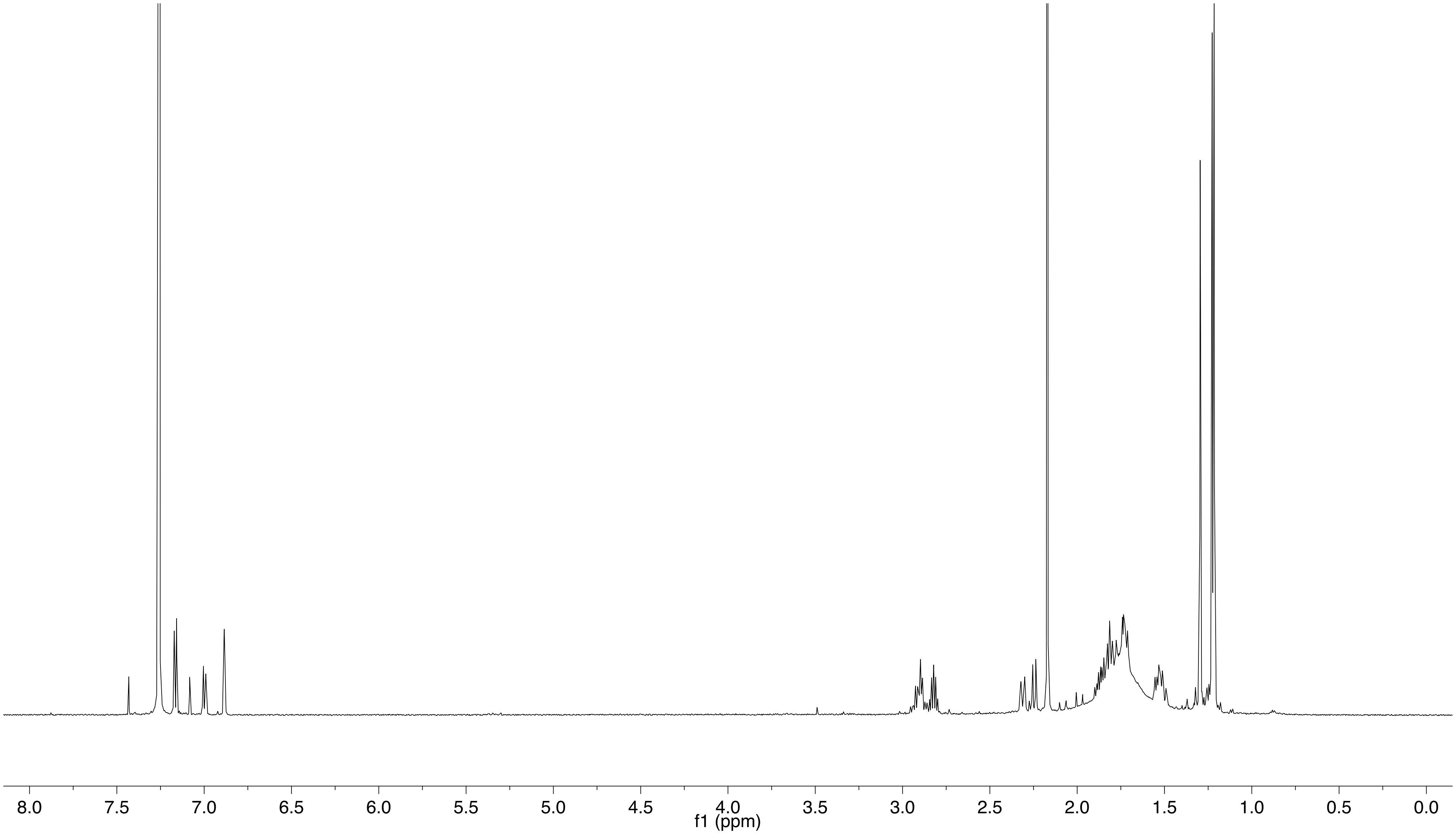

Fig. S11 – ^{1}H NMR (600 MHz, $CDCl_3$) spectrum of compound **1** isolated from strain LEGE 06105.

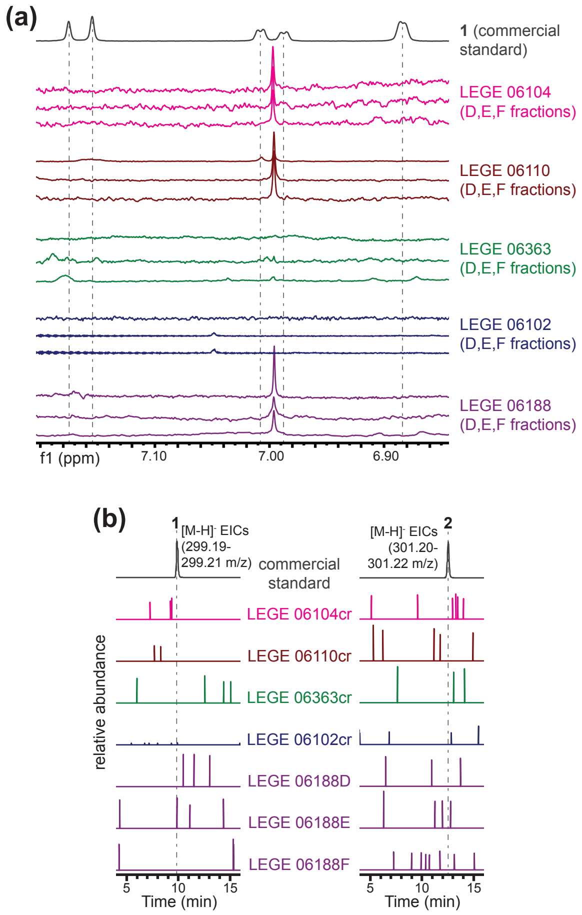

Fig. S12 – Compounds **1** or **2** were not detected by NMR and LC-HRESIMS in some cyanobacterial strains. a) ^{1}H NMR analysis of VLC fractions eluting with 50, 60 and 80% EtOAc in hexane (D, E and F, respectively and from top to bottom), with the region where the aromatic protons in 1 resonate (CDCl3) depicted. All spectra in $CDCl_3$, except for LEGE 06102D (acetone-*d6*). b) LC-HRESIMS analysis of crude fractions (dor LEGE 06104, LEGE 06110, LEGE 06363 and LEGE 06102) or D, E and F fractions (for strain LEGE 06188). Extacted Ion Chromatograms (EICs) for **1** and **2** are shown for each sample. Relative abundance is plotted for each EIC.

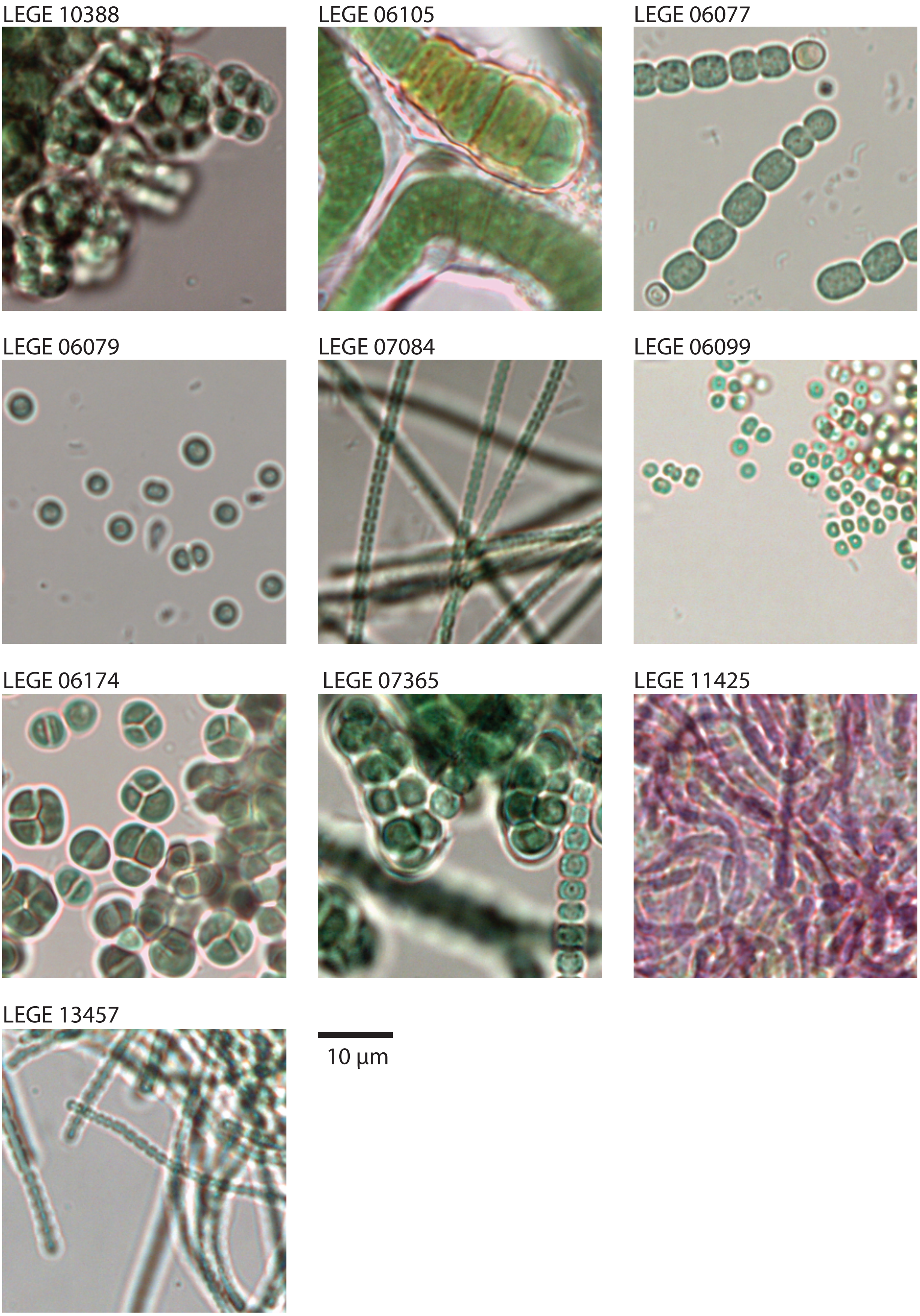

Fig. S13 – Optical microphotographs of the cyanobacterial strains used in this study that were found to produce metabolite **1**.

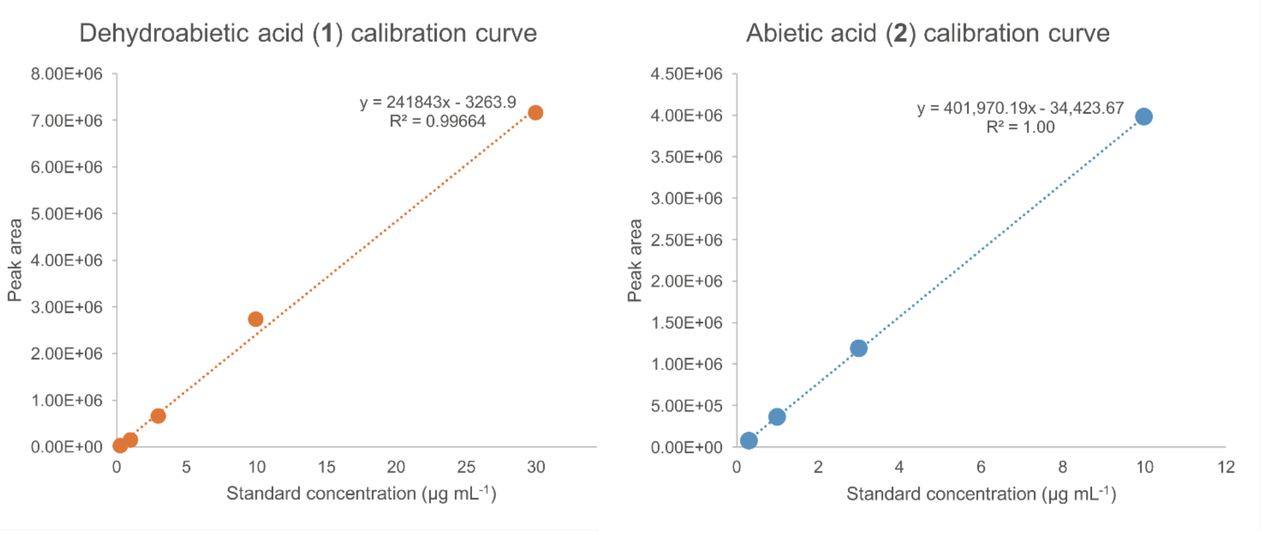

Fig. S14 – LC-HRESIMS calibration curves used to estimate the concentrations of the resin acids **1** and **2** in cyanobacterial cells and cyanobacterial culture supernatants. Calibration curve for **1** (left) and **2** (right) obtained using dilutions of commercial standards of the metabolites in MeOH.

Table S1 – Cyanobacterial homologs (from blastp, E-value $<1\times10^{-5}$ cut-off) of an abietadiene synthase (AAK83563.1) from *Abies grandis*

Homolog Accession No.	Annotated function	Organism	E-value	% coverage	%identity	%positives
WP_041033013	Hypothetical protein	*Tolypothrix campylonemoides*	2e-24	39	26	46
WP_017749312	Hypothetical protein	*Scytonema hofmanni*	3e-21	36	26	46
WP_048868993	Hypothetical protein	*Scytonema tolypothricoides*	3e-20	41	24	45
WP_017745060	Hypothetical protein	*Scytonema hofmanni*	4e-19	35	25	45
WP_038019343	Hypothetical protein	*Synechococcus* sp. PCC 7335	3e-06	34	26	44

Table S2 – Genomic context of the abietadiene synthase homolog (WP_041033013) from *Tolypothrix camylonemoides*

Relative position	Accession No	Annotated function	Top blastp hit with annotated function							Proposed function
			Accession No.	Organism	Annotated function	E-value	% coverage	% identity	% positives	
-4	WP_052490134	chromosome partitioning protein ParA	WP_048867650	*Scytonema tolypothrichoides*	plasmid partitioning protein ParA	1e-132	97	87	92	Chromosome partiotioning
-3	WP_041033016	transposase	WP_015211376	*Cylindrospermum stagnale*	transposase	0.0	99	84	89	transposase
-2	WP_041033015	short-chain dehydrogenase	WP_038086433	*Tolypothrix bouteillei*	short-chain dehydrogenase	0.0	100	88	94	SDR-family oxidoreductase
-1	WP_041033014	hypothetical protein	*No homologs found*							Hypothetical protien
+1	WP_052490133	hypothetical protein	WP_012234825	*Sorangium cellulosum*	cytochrome P450 CYP262A1	2e-137	90	48	68	Cytochrome P450
+2	WP_041033012	hypothetical protein	AHY18955	*Streptomyces platensis*	*ent*-kaurene synthase	0.014	80	23	41	*ent*-kaurene synthase-like lyase
+3	WP_041033011	tetratricopeptide domain protein	WP_048868991	*Scytonema tolypothrichoides*	tetratricopeptide domain protein	0	100	85	93	CHAT and tetratricopeptide domain protein
+4	WP_052490191	hemolysin activation/secretion protein	KIJ79289	*Tolypothrix campylonemoides* VB511288	hemolysin activation/secretion protein	0	98	99	100	Hemolysin activation/secretion protein

Supplementary Methods - Database searches for abietadiene synthase homologs in cyanobacterial genomes

The 853 amino acid sequence of abietadiene synthase from *Abies grandis* (AAK83563) was used as a query in a blastp search against the sequences in the NCBI database corresponding to Cyanobacteria (TaxID: 1117) on October 12th, 2015. Five hits were obtained with E-value < 1×10^{-5} (Table S1) corresponding to hypothetical proteins from strains belonging to the genera *Tolypothrix*, *Scytonema* and *Synechococcus*. Overall coverage was low (34-41%) and within matching regions identity ranged from 24 to 26% and similarity from 44-46%.

Closer inspection of the genome context of the top blastp hit (WP_041033013, from *Tolypothrix campylonemoides*) reveals some other biosynthesis-related functions (Table S2). These include a putative *ent*-kaurene synthase homolog, a cytochrome P450 homolog and a putative oxidoreductase, which, together with the abietadiene synthase homolog, could be hypothesized to participate in the biosynthesis of a tricyclic terpenoid such as **1** or **2**. Nevertheless, the low coverage or homology values do not allow to support some of the predicted functions.
